# Suicide ideation and attempts among people with epilepsy in Addis Ababa, Ethiopia

**DOI:** 10.1186/s12991-018-0174-6

**Published:** 2018-01-24

**Authors:** Kelelemua Haile, Tadesse Awoke, Getnet Ayano, Minale Tareke, Andargie Abate, Mulugeta Nega

**Affiliations:** 1 Department of Psychiatry, Amanuel Mental Specialized Hospital, Addis Ababa, Ethiopia; 2 Department of Epidemiology and Biostatistics, University of Gondar, Gondar, Ethiopia; 3 College of Medicine and Health Science, Bahir Dar University, Bahir Dar, Ethiopia; 4 College of Medicine and Health Science, Haramaya University, Harer, Ethiopia

**Keywords:** Epilepsy, Suicidal ideation, Suicidal attempt

## Abstract

**Background:**

Suicidal ideation and attempts are more frequent in people with epilepsy than in general population and suicide attempt increases the chance of later completed suicide. The aim of this study was to assess the prevalence and associated factors of suicidal ideation and attempt among people with epilepsy in Amanuel Mental Specialized Hospital, Addis Ababa, Ethiopia.

**Methods:**

Institution-based cross-sectional study was conducted from May to June 2014 at Amanuel Mental Specialized Hospital among people with epilepsy. The pre-tested semi-structured questionnaire was used for interviewing the study participants. Logistic regression analysis was used to assess predictors of suicidal ideation and attempt.

**Results:**

The study indicated that the prevalence of suicidal ideation and attempt among people with epilepsy were 29.8 and 14.1%, respectively. Poor social support, drug treatment for mental illness, had co-morbid depression, no seizure free within 1 year and family history committed suicide were significantly associated with suicidal ideation and attempt.

**Conclusion:**

The prevalence of suicidal ideation and attempt in people with epilepsy found to be higher when compared to general population. Therefore, screening all epilepsy patients should be done for early diagnosis and treatment.

## Introduction

Diagnostic and statistical manual of mental disorders, fifth edition (DSM-5) defines suicidal ideation as thoughts about self-harm with deliberate consideration or planning of possible techniques of causing one’s own death, while suicide is the act of intentionally causing one’s own death and suicide attempt is an attempt to end one’s own life, which may lead to one’s death [1]. There is a big difference between thinking about suicide and acting it out. Some persons may have ideas of suicide, but they will never act on. Some plan for days, weeks, or even years before acting, whereas others take their lives seemingly on impulse without advance planning [2].

Fifty percent of all violent deaths in men and 71% of women were accounted for suicides globally. Suicide rates are highest in persons aged 70 and older years for both men and women in almost all regions of the world [3]. Every year, more than 800,000 people die due to suicide (one person every 40 s) ranking as the second leading cause of death next to traffic accidents among 15–29 years of age [3].

The burden of suicide constitutes a serious public health issue worldwide that needs mental health professionals increase their awareness towards suicide warning signs. Suicide warning signs are associated with acute factors that inform clinicians about observable signs, expressed emotions, and important for saving lives by early detection and intervention for those at risk [4].

Epilepsy is a chronic neurological disorder affecting people of all ages, race and social class with more than 50 million global distribution [5]. It is commonly associated with brain dysfunction, social isolation and vocational difficulty making it a complex disorder [6]. Living with epilepsy affects relationships with family and friends, school, employment and leisure activities. Each of these effects may contribute to the high magnitude of psychiatric illness among people with epilepsy [7]. Patients with epilepsy have a higher risk of suicide compared to the general population giving that suicide is highly common co-morbid psychiatric illness [8].

Different studies indicated that people with epilepsy are at higher risk for suicidal thoughts and attempts [9, 10] with an estimated lifetime prevalence rate ranged from 3.3 to 14.3% [11] or even up to 35% [12]. This rate has been reported to be 6–25 times higher with temporal lobe epilepsy (TLE) compared to 1.4–6.9% in general population [13, 14].

Around 11% deaths in epilepsy are due to suicide, and a suicide attempt increased the chance of later completed suicide by 38% [15]. According to Centers for Disease Control and Prevention report, the suicide rate among people with epilepsy is 22% higher than the general population [16].

Despite this burden and consequences, there is a limited study on suicidal ideation and attempt in people with epilepsy in Ethiopia. Therefore, this study was intended to assess the magnitude and associated factors of suicidal ideation and attempt among people with epilepsy at Amanuel Mental Specialized Hospital.

## Methods

### Study settings and population

The institution-based cross-sectional study design was done from May to June 2014 at Amanuel Mental Specialized Hospital in Addis Ababa, Ethiopia. It is one of the oldest hospitals established in 1930E.C and the only mental Hospital in Ethiopia which is located in western part of Addis Ababa. The hospital has 255 beds and 18 outpatient departments that give serve for all types of mental disorder cases. Of which, two outpatient departments provide services for an average 2200 people with epilepsy monthly. People living with epilepsy (≥ 18 year) who have been clinically diagnosed with epilepsy and had follow-up treatment in outpatient epilepsy clinic in the Amanuel Mental Specialized Hospital were included in the study. However, patients unable to communicate and seriously ill were excluded from the study.

### Sample size and sampling procedures

Sample size was calculated using single population proportion formula $[ {{{n\, = \,\left( ({z {\alpha {/ 2} } } \right) 2 \, p \, ( {1 - p}))} / {d { 2} }}} ]$. By considering an assumption of 50% (0.5) proportion of suicidal ideation and attempt among people with epilepsy since it is unknown in our country, *Z*_*α*/2_ at 95% CI (1.96), and tolerable margin of error (0.05), the minimum sample size was 384. After adjusting for 10% contingency for non-response rate, a total of 423 study populations were involved in the study.

Sampling interval was determined by dividing total study population who had follow up during 1-month data collection period (2200) by total sample size (423). The sampling fraction is: 2200/423 ≈ 5. Hence, the sample interval is 5. The first study participant was selected by lottery method and the next study participants were chosen at regular intervals (every 5th) and interviewed by data collectors.

### Data collection and quality assurance

Data were collected by interviewing patients and reviewing charts using semi-structured questionnaire. World health organization composite international diagnostic interview (CIDI) was used to assess suicidal ideation and attempt among people with epilepsy [17]. Depression and social support were assessed using patient health questionnaire (PHQ-9) [18] and Oslo-3-item, respectively. Probable depression symptoms (PHQ-9 score ≥ 5) [19] and the individual who scored greater than or equal to 9 on Oslo 3 item consider as good social support [20]. Data were collected by four psychiatric nurses for 1-month period.

Semi-structured questionnaire for socio-demographic data and clinical related variables were developed in English and translated to local language (Amharic) to be understandable by all participants and translated back to English again to ensure its consistency. Training was given for four data collectors and one supervisor for 2 days. Pre-test was done at black lion hospital 2 weeks before the beginning of actual data collection. The data collectors were supervised daily and the filled questionnaires were checked properly by the supervisor and principal investigator to ensure its completeness.

### Data management and processing

The coded data were checked, cleaned and entered into epi.info version 3.5 and then exported into Statistical Package for the Social Sciences (SPSS) window version 20 for analysis. Descriptive statistic was used to explain the study participants in relation to study variable. Bivariate and multivariate logistic regression analyses were conducted to identify associated factors of suicidal ideation and attempt. The strength of the association was interpreted using odds ratio and 95% CI, and *p* value less than 0.05 was considered as statistically significant.

### Ethical consideration

Ethical clearance was obtained from the Institutional Review Board (IRB) of the College of Medicine and Health Sciences, University of Gondar, and from Amanuel Mental Specialized Hospital. The data collectors had clearly explained the aims of the study for study participants. We obtained written consent from each participant. The right was given to the study participants to refuse or discontinue participation at any time. Confidentiality was maintained throughout the study. Those study participants suffering from recurrent severe suicidal thought were treated by communicating with case team.

## Results

### Descriptions of socio-demographic characteristics of the respondents

A total of 410 respondents were enrolled and participated in the study which yields the response rate of 97%. The mean (± SD) age of respondents was 32.95 (± 11.87) year. There were more males 245 (59.8%) than females 165 (40.2%) (Table 1).

**Table 1:** Distribution of people with epilepsy disorder by their socio-demographic characteristics

Variables	Frequency (*n* = 410)	Percent (%)
Sex		
Male	245	59.8
Female	165	40.2
Age group		
18–24	110	26.8
25–31	113	27.6
32–38	68	16.6
39–45	59	14.4
> 45	60	14.6
Occupation		
Government employee	80	19
Merchant	73	17.8
Farmer	77	18.8
Student	52	12.9
Daily laborer	72	17.8
House wife	56	13.7
Educational level		
No education	61	14.9
Primary	178	43.4
Secondary	130	31.7
Diploma and above	41	10.0
Income (ETB*)		
< 750	254	62
750–1199	81	19.7
≥ 1200	75	18.3
Marital status		
Single	206	50.2
Married	141	34.4
Divorce/widowed	63	15.4
Living arrangement		
With family	351	85.6
Alone	59	14.4
Social support		
Poor	166	40.5
Good	244	59.5

ETB*, Ethiopian Birr

### Clinical characteristics and substance use

Regarding the onset of illness, 218 (53.2%) of the respondents were 18 years and above. Out of the total study participants, 18 (4.4%) and 19 (4.6%) had a family history of suicidal attempt and committed suicide, respectively (Table 2).

**Table 2:** Frequency distribution of clinical factors

Variables	Frequency (*n* = 410)	Percentage (%)
Age on set of epilepsy		
Under 18	192	46.8
18 and above	218	53.2
Duration of treatment (years)		
Up to 1	45	11.0
1–6	152	37.1
7–12	107	26.0
More than 12	106	25.9
Duration of illness (years)		
Up to 5	167	40.8
6–10	116	28.5
11–15	53	12.9
16–20	43	10.5
More than 20	30	7.3
Drug control on AED		
Seizure free/year	272	66.3
No seizure free/year	138	33.7
Co-morbid medical illness		
Yes	12	2.9
No	398	97.1
Drug taking for mental illness		
Yes	28	6.8
No	382	93.2
Co-morbid depression status		
Yes	116	28.3
No	294	71.7
Family history of epilepsy		
Yes	55	13.4
No	355	86.6
Family history of attempted suicide		
Yes	18	4.4
No	392	95.6
Family history of committed suicide		
Yes	19	4.6
No	391	95.4
Ever use substance		
Yes	129	31.4
No	281	68.6
Current substance use		
Yes	83	20.2
No	327	79.8

### Prevalence of suicidal ideation and suicidal attempt

The lifetime prevalence of suicidal ideation among respondents was 122 (29.8%); of whom, 80 (65.6%) reported suicidal ideation in less than 12 months and 73 (17.8%) had planned to commit suicide. Regarding suicidal attempt, the lifetime prevalence of suicidal attempt in this study was 58 (14.1%). Out of those who attempt suicide, 50 (86.2%) report to have suicidal attempt within the last 12 months and 36 (63.2%) of them attempt suicide once in their life (Table 3).

**Table 3:** Frequency distribution of life time prevalence suicide ideation and attempt

Variable	Frequency (*n* = 410)	Percentage (%)
Ever suicidal ideation		
Yes	122	29.8
No	288	70.2
Duration of ever seriously thought suicide		
≤ 12	80	65.6
> 12	42	34.4
Suicidal thought in 1 month		
Yes	30	7.3
No	380	92.7
Ever plan of suicide		
Yes	73	17.8
No	337	82.2
Duration of suicidal plan		
≤ 12	58	79.4
> 12	15	20.6
Suicidal attempt		
Yes	58	14.1
No	352	85.9
Duration ever suicidal attempt		
≤ 12	50	86.2
> 12	8	13.7
Number of suicide attempt		
One	36	62.1
Two	16	27.6
More than two	6	10.3
Reason for suicide Attempt		
Family conflict	33	31.3
Economic problem	18	16.5
Related to current illness	33	31.3
Death of family	11	10.1
Physical illness	2	1.8
Relate to hopelessness	12	11

**Fig. 1:**
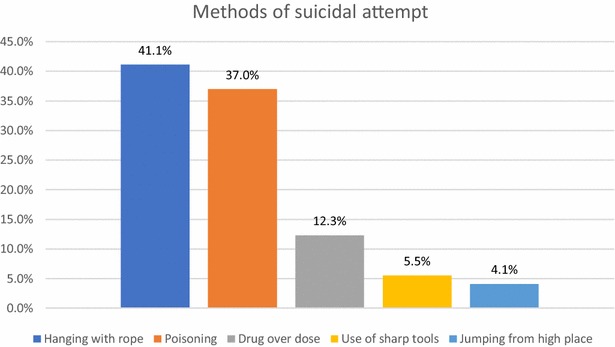
Methods of suicide attempt used among epilepsy patients

Different methods were used to attempt suicide (Fig. 1)

### Factors associated with suicidal ideation among people with epilepsy

The result of multivariate logistic regression revealed that those who live alone were 3.2 times more likely to have suicidal ideation than those who live with family (AOR 3.16, 95% CI 1.54, 6.46).

Respondents who had poor social support were 3.3 times more likely to have suicidal ideation as compared to those who had good social support (AOR 3.28, 95% CI 190, 5.68). In addition, those who had co-morbid depressive symptoms were 5.5 times more likely to have suicidal ideation compared to those who had no co-morbid depressive symptoms [AOR 5.47, 95% CI (3.12, 9.62)]. Taking drug treatment for mental illness also had a significant effect on suicidal ideation, indicating that those who were taking treatment were 4.2 times more likely to have suicidal ideation than those who had no history of mental illness and drug treatment for mental illness (AOR 4.16, 95% CI 1.42, 12.24). On the other hand, participants with no seizure free within 1 year were 2.6 times more likely to have suicidal ideation as compared to those with seizure free within 1 year (AOR 2.62. 95% CI 1.51, 4.56).

Concurrently, respondents who report family history of suicidal attempt were 4.4 times more likely to have suicidal ideation when compared to those who did not report a family history (AOR 4.36 95% CI 1.07, 17.80) (Table 4).

**Table 4:** Bivariate and multivariate analysis between some of selected factors and suicidal ideation

Explanatory variables	Suicide ideation		Crude OR (95% CI)	Adjusted OR (95% CI)
	Yes	No		
Sex				
Male	62	183	1.00	1.00
Female	60	105	1.69 (1.12, 2.59)	1.46 (0.78, 2.12)
Living arrangement				
Family	91	260	1.00	1.00
Alone	31	28	3.20 (1.585, 7.56)	3.16 (1.54, 6.46)*
Marital status				
Married	35	125	1.00	1.00
Single	66	140	1.684 (1.046, 2.709)	0.64 (0.28, 1.46)
Separate/divorced/widowed	21	23	3.261 (1.619, 6.569)	1.33 (0.59, 3.02)
Social support				
Good	41	203	1.00	1.00
Poor	81	85	4.72 (2.99, 7.42)	3.28 (1.90, 5.68)*
Co-morbid depressive symptoms				
Yes	75	41	9.61 (5.877, 15.73)	5.47 (3.12, 9.62)*
No	47	247	1.00	1.00
Family history of attempted suicide				
Yes	14	4	9.20 (2.96, 28.57)	4.36 (1.07, 17.80)*
No	108	284	1.00	1.00
Drug control on AED				
Seizure free/year	55	217	1.00	1.00
No seizure free/year	67	71	3.72 (2.38, 5.82)	2.62 (1.51, 4.56)*
Drug taking for mental illness				
Yes	19	9	5.72 (2.51, 13.04)	4.16 (1.42, 12.24)*
No	103	279	1.00	1.00

* *p* value < 0.05

### Factor associated with suicidal attempt among people with epilepsy

The result of multivariate logistic regression model revealed that clients who had poor social support, those on drug treatment for mental illness, had co-morbid depressive symptoms, no seizure free within 1 year and family history committed suicide were significantly associated with suicidal attempt (Table 5).

**Table 5:** Bivariate and multivariate logistic regression analysis between some of selected factors and suicidal attempt

Explanatory variables	Suicide attempt		COR	AOR
	Yes	No		
Sex				
Male	27	218	1.00	
Female	31	134	1.87 (1.07, 3.27)	1.63 (0.76, 3.51)
Living arrangement				
Family	44	307	1.00	
Alone	14	45	2.17 (1.10, 4.28)	1.02 (0.38, 2.76)
Social support				
Yes	16	228	1.00	1.00
No	42	124	4.83 (2.61, 8.94)	3.48 (1.96.6.16)*
Co-morbid depressive symptoms				
Yes	45	71	13.70 (7.01, 26.77)	7.84 (3.58, 15.21)*
No	13	281	1.00	1.00
Family history committed suicide				
Yes	10	9	7.94 (3.07, 20.53)	5.32 (1.55, 18.20)*
No	48	343	1.00	1.00
Drug taking for mental illness				
Yes	16	12	10.79 (4.78, 24.37)	6.81 (3.00, 22.45)*
No	42	340	1.00	1.00
Drug control on AED				
Seizure free/year	22	250	1.00	1.00
No seizure free/year	36	102	4.01 (2.25, 7.15)	3.19 (1.48, 6.86)*

* *p* value < 0.05

## Discussion

In this study, the prevalence of lifetime suicidal ideation and attempt among people living with epilepsy and their possible associations with different variables were assessed. The prevalence of suicidal ideation was 29.8% which is higher than the result reported in Egypt (23.5%) [12], in Washington tertiary epilepsy clinics (11.9%) [21]. These might be due to the difference in sample size, study design, study participants, culture, time variation, and settings. In addition, in Washington, it was current suicide ideation but this study was lifetime prevalence.

However, the current finding is lower than from Bosnia and Herzegovina reported (38%) [22], Brazil (36.7%) [23], Cuba Havana (45.2%) [24]. The discrepancy might be due to the difference in settings, sample size, and study participants. The other possible reason might be the difference in study design since we used institution-based cross-sectional study design, but Brazilian study was community-based case–control study. Furthermore, in Cuba Havana, study subjects were patients with temporal lobe epilepsy, but our study included all people living with epilepsy.

Regarding suicidal attempt, the current study found that the prevalence of lifetime suicidal attempt among people with epilepsy was 14.1% which is closely consistent with many other reports in Egypt (11.5%) [12], Croatia (14.6%) [25], and Brazil (12.1%) [26]. However, this result was higher than the study done in Bosnia and Herzegovina (18%) [22], Cuba Havana (28.6%) [24]. The difference might be due to sample size, study participants, and study design described above.

The most commonly used method for suicidal attempt in people with epilepsy in this study was hanging (41%) which is inconsistent with the study findings from different countries. For instance, 34.9% of Korean [27] and 87.5% of Japanese [28] study participants used drug overdose especially Phenobarbital. This discrepancy might be due to cultural difference, availabilities of methods and knowledge of participants. Poisoning by pesticide is common in many Asian countries and in Latin America while poisoning by drugs is common in northern Europe countries and the United Kingdom. Hanging is the preferred method of suicide in Eastern Europe and using gun shooting is common in the United States and jumping from a high place in cities and urban societies such as Hong Kong Special Administrative Region, China [29].

In this study, those participants living alone were more likely to have suicidal ideation. The possible reason could be those who live alone could not share the problem nearby family on time; this increases hopelessness and may lead to suicidal ideation which is supported by study done in Washington [21].

Those respondents who had poor social support, no seizure free within 1 year were predictors for suicidal ideation in this study. The previous study done in Ethiopia revealed that frequent seizure attacks were associated with depression and increased perceived stigma [30, 31]. This may, in turn, increase suicidal ideation and attempt in people with epilepsy. WHO report in 2004 showed that weak social ties and low support from friends or relatives have been significantly associated with suicidal ideation [32]. The reason may be repeated seizure attack, increased lesion in the brain with neuron-chemical involvement, and the increased frequency could be again low coping mechanism contributing to suicidal ideation [12].

Those study participants who were taking drug for mental illness in addition to epilepsy drug and having co-morbid depressive symptoms were highly exposed for suicidal ideation. This was in line with study done in Denmark, Sweden, Croatia and Washington [25, 33, 34]. The possible reason could be presence of mental illness by itself and drug treatment for longer time may make negative view of life.

Respondents who had family history of suicidal attempt were found to have suicidal ideation. This was in line with the study in Chicago and Denmark [15, 35]. The possible reason might be from biological perspective; environmental and non-genetic such as shared exposure to the family stress and common life style could contribute to the suicidal ideation.

Furthermore, social support and co-morbid depressive symptoms among people with epilepsy were significantly associated with suicidal attempt which is consistent with study done in Finland, Japan and Cuba [28, 36]. These factors might be due to misunderstanding of the disorders, avoidance from family and workplace lead to unemployment, poor social ties and low support increases patients’ suicidal attempt. Depression increase suicidality due to the effect in neuron-transmitter alteration in people with epilepsy [37]. On another hand, those depressed patients have hopelessness and suicidal ideation making them attempt suicide [38].

Participants having drug treatment for mental illness and no seizure free within 1 year were associated with suicidal attempt which is in line with study done in Sweden and Egypt [12, 34]. Since the presence of mental illness like mood disorder, schizophrenia and anxiety can increase suicidality. Some psychotropic drugs may lower seizure threshold which makes it easier for patient to experience seizure [39]. The co-existing of two chronic illnesses and drug treatment for longer time may make negative attitude for life leading to suicidal attempt. Fear of having seizure attack in public place can affect their performance and contribute to poor self-esteem, social isolation, negatively influence on their ability to work and finally may result in suicidal attempt [40].

It is the first study in Ethiopia that determined the prevalence and associated factors for both suicidal ideation and attempt. However, the discussion was done by considering the limitations of not addressing types of medication and types of epilepsy because it was difficult to get specific diagnosis in the patient’s chart.

## Conclusion

The prevalence of suicidal ideation and attempt among people living with epilepsy were found to be high. Social support, living alone, co-morbid depression, partially controlled seizure, drug taking for mental illness, family history of suicidal attempt and committed suicide were significantly associated with suicidal ideation and attempt independently. Screening of suicidal ideation and attempt for all epilepsy patients should be done for early diagnosis and treatment. It is better to conduct a further longitudinal study among epilepsy and co-morbid mental illness with their specific drug treatment for suicidal ideation and attempt.

## References

[1] American Psychiatric Association (2013). Diagnostic and statistical manual of mental disorders.

[2] Sadock BJ, Sadock VA (2015). Kaplan & Sadock’s synopsis of psychiatry: behavioral sciences/clinical psychiatry.

[3] World Health Organization (2014). Preventing suicide. A global imperative.

[4] Rudd MD (2008). Suicide warning signs in clinical practice. Curr Psychiatry Rep.

[5] World Health Organization (2010). Epilepsy in the WHO eastern Mediterranean region: bridging the gap.

[6] Mazza M, Bria P, Mazza S (2007). Depression and suicide in epilepsy: a fact or artifact?. J Neurol Sci.

[7] Alsaadi T, Zamel K, Sameer A, Fathalla W, Koudier I (2013). Depressive disorders in patients with epilepsy: why should neurologists care?. Health.

[8] Thompson AW, Miller JW, Katon W, Chayto N, Ciechanowski P (2009). Sociodemographic and clinical factors associated with depression in epilepsy. Epilepsy Behav.

[9] Meador KJ (2008). Suicide in patients with epilepsy. Epilepsy Curr.

[10] Pack AM (2016). Epilepsy and suicidality: what’s the relationship?. Epilepsy Curr.

[11] Jones JE, Hermann BP, Barry JJ, Gilliam FG, Kanner AM, Meador KJ (2003). Rates and risk factors for suicide, suicidal ideation, and suicide attempts in chronic epilepsy. Epilepsy Behav.

[12] Hamed SA, Elserogy YB, Abdou MA, Abdellah MM (2012). Risks of suicidality in adult patients with epilepsy. World J Psychiatry.

[13] Jallon P (2004). Mortality in patients with epilepsy. Curr Opin Neurol.

[14] Christensen J, Vestergaard M, Mortensen P, Sidenius P, Agerbo E (2007). Epilepsy and risk of suicide: a population-based case–control study. Lancet Neurol.

[15] Kanner AM (2009). Suicidality and epilepsy: a complex relationship that remains misunderstood and underestimated. Curr Rev Clin Sci.

[16] CDC (2016). Suicide rate is 22% higher among people with epilepsy than the general population. Epilepsy Behav.

[17] Kessler RC, Üstün TB (2004). The world mental health (WMH) survey initiative version of the world health organization (WHO) composite international diagnostic interview (CIDI). Int J Methods Psychiatr Res.

[18] Gelaye B, Williams MA, Lemma S, Deyessa N, Bahretibeb Y, Shibre T, Wondimagegn D, Lemenhe A, Fann JR, Vander Stoep A (2013). Validity of the patient health questionnaire-9 for depression screening and diagnosis in East Africa. Psychiatry Res.

[19] Bitew T, Hanlon C, Kebede E, Honikman S, Fekadu A (2017). Antenatal depressive symptoms and perinatal complications: a prospective study in rural Ethiopia. BMC Psychiatry.

[20] Meltzer H, Nosikov A, Gudex C (2003). Development of a common instrument for mental health. EUROHIS: developing common instruments for health surveys.

[21] Hecimovic H, Santos JM, Carter J, Attarian HP, Fessler AJ, Vahle V, Gilliam F (2012). Depression but not seizure factors or quality of life predicts suicidality in epilepsy. Epilepsy Behav.

[22] Andrijić NL, Alajbegović A, Zec SL, Loga S (2014). Suicidal ideation and thoughts of death in epilepsy patients. Psychiatr Danub.

[23] Stefanello S, Marín-Léon L, Fernandes PT, Li LM, Botega NJ (2010). Psychiatric comorbidity and suicidal behavior in epilepsy: a community-based case–control study. Epilepsia..

[24] Espinosa AG, Machado RA, González SB, González ME, Montoto AP, Sotomayor GT (2010). Wisconsin Card Sorting Test performance and impulsivity in patients with temporal lobe epilepsy: suicidal risk and suicide attempts. Epilepsy Behav.

[25] Buljan R, Šantić AM (2011). Suicide attempts in hospital-treated epilepsy patients. Acta Clin Croat.

[26] Salgado PCB, Nogueira MH, Yasuda CL, Cendes F (2012). Screening symptoms of depression and suicidal ideation in people with epilepsy using the beck depression inventory. J Epilepsy Clin Neurophysiol.

[27] Seo JG, Lee JJ, Cho YW, Lee SJ, Kim JE, Moon HJ, Park SP (2015). Suicidality and its risk factors in Korean people with epilepsy: a MEPSY study. J Clin Neurol.

[28] Hara E, Akanuma N, Adachi N, Hara K, Koutroumanidis M (2009). Suicide attempts in adult patients with idiopathic generalized epilepsy. Psychiatry Clin Neurosci.

[29] Ajdacic-Gross V, Weiss MG, Ring M, Hepp U, Bopp M, Gutzwiller F, Rössler W (2008). Methods of suicide: international suicide patterns derived from the WHO mortality database. Bull World Health Organ.

[30] Tegegne MT, Mossie TB, Awoke AA, Assaye AM, Gebrie BT, Eshetu DA (2015). Depression and anxiety disorder among epileptic people at Amanuel Specialized Mental Hospital, Addis Ababa, Ethiopia. BMC Psychiatry.

[31] Tegegne MT, Awoke AA. Perception of stigma and associated factors in people with epilepsy at Amanuel Specialized Mental Hospital, Addis Ababa, Ethiopia. Int J Psychiatry Clin Pract. 2016;21(1):58–63. 10.1080/13651501.2016.1223315 27626512

[32] World Health Organization (2004). Suicide huge but preventable public health problem.

[33] Christensen J, Vestergaard M, Mortensen PB, Sidenius P, Agerbo E (2007). Epilepsy and risk of suicide: a population-based case–control study. Lancet Neurol.

[34] Nilsson L, Ahlbom A, Farahmand BY, Asberg M, Tomson T (2002). Risk factors for suicide in epilepsy: a case control study. Epilepsia.

[35] Qin P, Agerbo E, Mortensen PB (2002). Suicide risk in relation to family history of completed suicide and psychiatric disorders: a nested case–control study based on longitudinal registers. Lancet.

[36] Mainio A, Alamäki K, Karvonen K, Hakko H, Särkioja T, Räsänen P (2007). Depression and suicide in epileptic victims: a population-based study of suicide victims during the years 1988–2002 in northern Finland. Epilepsy Behav.

[37] Kanner AM (2005). Depression in epilepsy: a neurobiologic perspective. Epilepsy Curr.

[38] Hawton K, i Comabella CC, Haw C, Saunders K (2013). Risk factors for suicide in individuals with depression: a systematic review. J Affect Disord.

[39] Spina E, Trifirò G, Caputi AP. Pharmacovigilance in psychiatry: an introduction. In: Pharmacovigilance in psychiatry. Springer; 2016. p. 3–7.

[40] Pompili M, Vanacore N, Macone S, Amore M, Perticoni G, Tonna M, Sasso E, Lester D, Innamorati M, Gazzella S (2007). Depression, hopelessness and suicide risk among patients suffering from epilepsy. Ann Ist super sAnItà 2007.

