# Correction to: Suicide ideation and attempts among people with epilepsy in Addis Ababa, Ethiopia

**DOI:** 10.1186/s12991-018-0182-6

**Published:** 2018-03-07

**Authors:** Kelelemua Haile, Tadesse Awoke, Getinet Ayano, Minale Tareke, Andargie Abate, Mulugeta Nega

**Affiliations:** 1 Department of Psychiatry, Amanuel Mental Specialized Hospital, Addis Ababa, Ethiopia; 2 Department of Epidemiology and Biostatistics, University of Gondar, Gondar, Ethiopia; 3 College of Medicine and Health Science, Bahir Dar University, Bahir Dar, Ethiopia; 4 College of Medicine and Health Science, Haramaya University, Harer, Ethiopia

## Correction to: Ann Gen Psychiatry (2018) 17:4 10.1186/s12991-018-0174-6

After publication of the article [1], it has been brought to our attention that an author’s name was spelt incorrectly in the original published article. Getinet Ayano was previously spelt “Getnet Ayano”. This has now been corrected in the revised version of the article.
